# Supplementary material for: Predictors of malaria Rapid Diagnostic Tests’ utilisation among healthcare workers in Zamfara State
Source: PLoS One. 2018 Dec 14;13(12):e0200856. doi: 10.1371/journal.pone.0200856 (PMC6294357; doi:10.1371/journal.pone.0200856)
Supplement: S1 Questionnaire — (DOC) [file pone.0200856.s001.doc]

**QUESTIONNAIRE ON FACTORS AFFECTING UTILIZATION OF MALARIA RAPID DIAGNOSTIC TESTS BY HEALTHCARE WORKERS IN ZAMFARA STATE**

*Questionnaire for Health Workers*

**Introduction and Consent**

Good morning Sir/Ma, I am an MPH student of Ahmadu Bello University, Zaria. I am working on a study related to the use of Malaria Rapid Diagnostic Tests (mRDTs). I am interested in understanding your experience with using RDTs. I would like to ask you some questions about your experiences and opinions as related to use of mRDTs. I would appreciate it if you can answer the questions as honestly and freely as you can. Whatever information you choose to share with me will be treated confidentially. If at any time you don’t want to answer a question, just tell me, and I will stop the interview or move to another question. Your responses will be analyzed together with those from some other health workers and will be used to make recommendations for further improvement in use of malaria RDTs.

Do you agree to the interview? Yes No

Date of Interview _______________________________

Name of interviewer ______________________________________

Questionnaire ID ______________

Name of Facility ______________________________________

Private Public

Primary Secondary

**Socio-demographic characteristics**

1. Sex: (a) Male (b) Female
2. Age as at last birthday (years) ______________
3. Marital status: (a) Married Single Divorced Widowed
4. Professional Cadre:

(a) CHEW (b) Nurse (c) Doctor (d) Lab. Scientist/technician (e) CHO

4b) Grade level/ Designation (e.g. CNO/ GL 14) _________________________

1. Highest Professional qualification of respondent ______________
2. No. of year(s) working as a health worker ______________

**Knowledge and Utilization of malaria RDT (mRDT)**

1. What does malaria RDT stand for? ______________
2. What does it assess? ______________
3. What type of specimen is used for malaria diagnosis when using mRDT? (do not read out the options) (a) Blood (b)Urine (c) Stool (d) Don’t know
4. How is the above specimen obtained? (do not read out options) (a) Needle prick (b)Venepuncture (c) Urinating into a bottle (d)Don’t know
5. How much blood is required to carry out mRDT? (do not read out options) (a) 5μL (b) 10 μL (c) 1mL (d)A drop (e) Don’t know
6. Do you know how to carry out an mRDT? (a) Yes (b)No

df

*(If No, skip to question 18)*

1. Does it take a long time to carry out? (a) Yes (b) No
2. On the average, how long does it take to carry out one mRDT? (in minutes) ______________

df

1. What tests are used to diagnose malaria in this facility? (a)mRDTs (b)Microscopy (c)Clinical diagnosis (d)mRDTs and microscopy (e) Others (specify)____________

df

df

df

1. If mRDT is not used, why? ________________________________________________________________________________________________________________________________________________________
2. Do you use the results of mRDT to determine who receives or does not receive ACT? (a)Yes (b) No

df

- df

1. (If answer to question 18 is Yes) **Why**?(more than one answer is possible)

(a) You are more confident of what you are treating

df

df

(b) It will prevent unnecessary use of ACTs

df

(c) Conditions other than malaria can be considered

(d) I have more confidence in the result than microscopy

df

(e) Other reasons (pls specify)________________________

1. (If answer to question 18 is No) **Why**?(more than one answer is possible)

(a) Test is not necessary

df

df

(b) I can diagnose malaria accurately without a test

df

(c) The test is not reliable

(d) Other reasons (pls specify)_____________________________

**Training**

df

1. Have you received training on malaria case management in the last 6 months? (a)Yes (b)No

df

*(If No, skip to question 24)*

1. If answer to question 21 is Yes, what type of training did you receive? *Tick all that are applicable*

df

1. Training on malaria case management mRDT inclusive

df

1. Training in mRDT only

(c) Training in malaria in pregnancy (MiP)

df

1. Who conducted the last training you attended on the use of mRDT?

df

1. Ministry of Health
2. Partner organization (e.g. WHO, UNICEF, MAPS, SHI,) *specify___________________*
3. Non-governmental organization, please*specify ___________________*

**Perception and diagnostic accuracy of RDTs**

df

df

1. Do you use mRDT routinely in this facility? (a)Yes (b) No
2. What do you think about the accuracy of malaria rapid diagnostic tests (mRDT)?

df

1. I fully trust the results of the mRDTs

df

1. I have some doubts about the results of the mRDTs
2. I don’t trust the results of the mRDTs

df

1. If you don’t trust the result of mRDTs, why?

_____________________________________________________________________

_____________________________________________________________________

1. Who performs the mRDT in your facility? *(you can choose one or more)*
2. Yourself

df

1. Laboratory Technician in your facility *(patient is referred to the lab)*

df

1. Another health worker in your facility

df

1. Done at a laboratory outside your facility

df

1. Others *specify* __________________

df

1. Do you wait for result of mRDT before you treat a suspected case of malaria? (a)Yes (b)No

df

df

df

1. Do you comply with the result? (a)Always (b) Sometimes (c) Never

df

df

(d) Rarely

1. Which drug do you use to treat a confirmed case of malaria? (a) ACTs (b) Chloroquine (c)Sulphadoxine-pyrimethamine (d)Others (please specify)_______________

df

df

df

1. Was the test difficult to carry out? (a)Yes (b)No

df

df

1. Have patients ever rejected having the test? (a) Yes (b)No

df

df

1. Have patients ever rejected having the test results? (a) Yes (b)No

df

df

df

1. Do the staff members consider the test time-consuming? (a)Yes (b)No

df

df

1. Did conducting mRDT interfere with clinic activities? (a)Yes (b) No

df

1. Do you think mRDT is better than other methods used to diagnose malaria? (a)Yes (b)No

df

df

If Yes, Why? _____________________________________________________________________

_____________________________________________________________________

If No, why? __________________________________________________________________

1. Are there still some challenges that you are experiencing with using mRDT result for managing fever? (a)Yes (b)No

df

df

1. If yes, what are the challenges? _____________________________________________________________________

_____________________________________________________________________

**Availability of mRDT**

1. Do you currently have mRDT in this facility? (interviewer check malaria RDT to verify) (a) Yes (b)No

df

1. Do you have the mRDT User’s Manual in your facility? (a)Yes (b)No

1. Has there been a regular supply of mRDT in this facility? (a)Yes (b)No

1. Have you experienced stock-out in this facility in the past 1 week? (a) Yes (b)No

**Supervision**

1. Have you ever received supportive supervision on case management of malaria?

(a)Yes (b) No

1. When did you receive the last supportive supervision on case management of malaria?

1. In the last 1 week

1. In the last 1 month
2. In the last 6 months

1. In the last 12 months

1. More than 12 months ago
2. Never did

1. Who conducted the last supportive supervision?

1. A senior officer from this facility
2. A senior officer from the Local Government

1. An officer from the state ministry of health
2. An officer from the federal ministry of health

1. An officer from the development partners *(e.g. WHO, MAPS....) Please specify* _____________________________
2. Others specify______________________
3. What activities did he/she carry out during supervision?

__________________________________________________________________________________________________________________________________________

1. Did the supervisor use a checklist? (a) Yes (b) No Don’t know

1. Did the supervisor give you a feedback (a)Yes (b) No

*If Yes to question 48, go to the next question (Q.49), if No go to question 50)*

1. Feedback was (a)Verbal (b)Written (c) Both verbal and written

**Laboratory Services**

1. How far is the nearest laboratory to this facility
2. Within the facility premises

1. Less than 1 km from facility

1. 1 to 5 km from the facility

1. More than 5km
2. How much does it cost to have malaria test in this laboratory?
3. Microscopy ________________
4. mRDT ___________________

*Thanks for your time and participation*
